# Morphometric and Histological Characterization of Chestnuts in Dezhou Donkeys and Associations with Phenotypic Traits

**DOI:** 10.3390/vetsci12090846

**Published:** 2025-09-01

**Authors:** Wenting Chen, Xiaotong Liu, Qifei Zhu, Junjie Liu, Abd Ullah, Yihong Liu, Jinjin Wei, Muhammad Zahoor Khan, Changfa Wang

**Affiliations:** College of Agriculture and Biology, Liaocheng University, Liaocheng 252000, China

**Keywords:** equine, donkey, skin, chestnut, histology, morphometry

## Abstract

Background: Chestnuts are keratinized skin structures found on equine limbs, but their characteristics in donkeys remain poorly understood. This study aimed to characterize chestnut morphology and histology in Dezhou donkeys and examine correlations with phenotypic traits. Methods: A cross-sectional study was conducted on 347 Dezhou donkeys (0.3–15 years, 79–419 kg). Chestnut dimensions were measured using precision calipers, and correlations were analyzed with age, body weight, limb measurements, and thoracolumbar vertebral counts. Histological analysis compared chestnut tissue with adjacent normal skin using standard H&E staining protocols. Results: Donkeys exclusively possessed chestnuts on forelimbs, predominantly showing regular geometric configurations. Histologically, chestnut tissue exhibited marked hyperkeratosis (>30 cellular layers vs. 4–6 in normal skin), widespread melanocyte distribution throughout the epidermis, and complete absence of cutaneous appendages. In group A, strong positive correlations were observed between chestnut width and age (r = +0.527, *p* < 0.01), body weight (r = +0.538, *p* < 0.01), and limb measurements (r > +0.589 *p* < 0.01). No significant correlations existed with vertebral numbers. In group B Dezhou donkeys older than 2 years, the length and width of the forelimb chestnuts showed the strongest significant correlation with right forelimb measurements, while no significant correlations were observed with other variables (age, body weight, and hindlimb measurements). Conclusions: Chestnuts in Dezhou donkeys represent specialized integumentary structures with unique histological features and strong correlations with somatic development. These findings support their potential utility as biometric markers for individual identification and indicate coordinated developmental regulation with overall growth patterns.

## Simple Summary

This study examined chestnuts (keratinized skin structures) in 347 Dezhou donkeys to understand their morphological characteristics and correlations with phenotypic traits. Unlike horses, donkeys only have chestnuts on their forelimbs. Histological analysis revealed that chestnut tissue differs significantly from normal skin, showing hyperkeratosis, increased melanocyte distribution, and absence of hair follicles and glands. It was found among Group A Dezhou donkeys aged 0–2 years that strong positive correlations were found between chestnut width and age (r = +0.527), body weight (r = +0.538) and limb measurements. No significant correlations existed with vertebral numbers. These findings suggest that chestnuts could serve as biometric markers for individual identification and indicate coordinated developmental programs with overall somatic growth.

## 1. Introduction

Chestnuts, also known as ergots, are distinctive keratinized integumentary structures found on the limbs of equids, representing one of the most intriguing yet understudied anatomical features in veterinary morphology [1]. These specialized skin appendages have garnered scientific attention primarily in horses, where they manifest on all four limbs and exhibit considerable morphological diversity [1,2]. However, despite their potential significance as morphometric markers and their unique developmental characteristics, chestnuts in donkeys (*Equus asinus*) remain remarkably understudied, representing a significant gap in our understanding of equid comparative anatomy.

In horses, previous investigations have established that chestnuts demonstrate species-specific distribution patterns and morphological characteristics that correlate with various phenotypic traits, including body weight and age [1,3]. These findings have prompted speculation about their potential utility as biometric identification tools and their role in understanding coordinated developmental processes. However, the anatomical distribution, histological architecture, and morphometric relationships of chestnuts in donkeys differ substantially from their equine counterparts, necessitating dedicated investigation.

The Dezhou donkey, a prominent Chinese breed valued for both agricultural work and meat [4] and hide production [5,6,7], presents an ideal model for investigating donkey chestnut characteristics due to its well-documented phenotypic traits and standardized breeding practices. Preliminary observations suggest that donkeys possess chestnuts exclusively on their forelimbs, contrasting sharply with the four-limb distribution observed in horses. This anatomical distinction, combined with the limited research on donkey integumentary structures, underscores the need for comprehensive morphometric and histological characterization.

Furthermore, the potential correlation between chestnut morphology and various phenotypic parameters—including age, body weight, limb dimensions, and skeletal configurations—remains unexplored in donkeys. Understanding these relationships could provide valuable insights into developmental biology, offer practical applications for animal identification and breeding programs, and contribute to the broader field of comparative equid morphology.

The present study addresses these knowledge gaps by conducting the first comprehensive morphometric and histological analysis of chestnuts in Dezhou donkeys. Our objectives were threefold: (1) to characterize the morphological and histological features of donkey chestnuts compared to adjacent normal skin; (2) to quantify correlations between chestnut dimensions and key phenotypic traits, including age, body weight, limb measurements, and vertebral configurations; and (3) to evaluate the potential utility of chestnuts as biometric markers for individual identification. Through systematic analysis of 347 Dezhou donkeys (82 male donkeys and 265 female donkeys) representing a broad age and weight range, this investigation provides novel insights into the specialized nature of these integumentary structures and their integration within coordinated developmental programs.

## 2. Materials and Methods

### 2.1. Study Design and Animal Selection

This cross-sectional study was conducted at three provincially authorized Dezhou donkey breeding farms in northwest Shandong Province, China. A total of 347 Dezhou donkeys, ranging in age from 0.3 to 15 years (mean ± SD: 4.89 ± 3.2 years) and weighing between 79 and 419 kg (mean ± SD: 257.41 ± 85.3 kg), were included in this study. The animals were divided into two groups based on their age: Group A, comprising 169 immature donkeys aged 0–2 years; and Group B, comprising 178 mature donkeys over 2 years of age. All animals were maintained under standardized management conditions with twice-daily feeding schedules and ad libitum access to water [8]. The study protocol was approved by the institutional Special Committee of Scientific Research Ethic of Liaocheng University (AP2025031726), and all procedures were conducted in accordance with established ethical guidelines for animal research.

### 2.2. Morphometric Measurements

#### 2.2.1. Chestnut Dimensional Analysis

**Figure 1:**
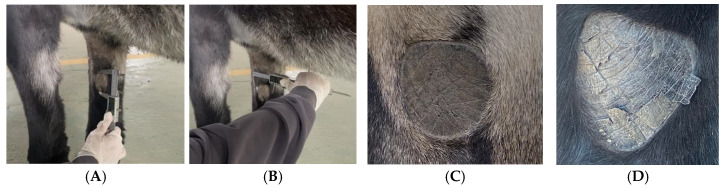
Chestnut dimension measurement. (**A**) Length measurement, (**B**) width measurement, (**C**) chestnut length–width view of Sanfen, (**D**) chestnut length–width view of Wutou.

Chestnut dimensions were measured using precision Vernier calipers (accuracy ± 0.1 mm). Chestnut length (CL) was defined as the distance between the superior and inferior margins, while chestnut width (CW) was measured as the distance between the lateral margins at the widest point. All measurements were performed by a single trained investigator to minimize inter-observer variability and were recorded in centimeters to one decimal place (Figure 1).

#### 2.2.2. Biometric Assessments

The weight of the animals was measured using a calibrated 1000 kg capacity electronic scale, and they were placed in a standardized standing position. No feeding was carried out before weighing. Pre-measurement calibration and zeroing procedures were performed, and readings were recorded when weight stabilized (±1 kg accuracy).

Limb height measurements were obtained using a flexible measuring tape. Forelimb height (FLH) was measured as the vertical distance from the shoulder joint to ground level, while hindlimb height (HLH) was measured from the tuber ischium to ground level [1]. All measurements were taken with animals standing on level ground in natural posture. Animal age was determined using birth records documented in the farm management system.

#### 2.2.3. Radiographic Vertebral Assessment

Thoracolumbar vertebrae enumeration was conducted using digital radiography (DR) systems (Liaoning Kaipu Medical System Co., Ltd., Benxi, Liaoning Province, China) [9]. Animals were positioned in lateral recumbency under appropriate sedation protocols. High-resolution radiographic images were obtained to facilitate accurate counting of thoracic vertebrae number (TVN) and lumbar vertebrae number (LVN). All radiographic interpretations were performed by qualified veterinary radiologists to ensure consistency and accuracy.

### 2.3. Histological Sample Collection and Processing

#### 2.3.1. Tissue Sample Acquisition

Tissue samples were collected from female donkeys (*n* = 2 mature adults, *n* = 1 male fetus at 7 months gestation from natural abortion) at Huimin Agricultural Science and Technology Co., Ltd., Yucheng, Shandong Province, China. Following standardized humane slaughter protocols, including electrical stunning and exsanguination via jugular venesection, 1 cm^2^ specimens were obtained using sterile biopsy punches (Hyde Entrepreneurship (Beijing) Biotechnology Co., Ltd., Beijing, China) from the left hindlimb chestnut tissue and adjacent normal skin located 5 cm proximal to the chestnut.

#### 2.3.2. Histological Processing Protocol

Tissue specimens were immediately fixed in neutral buffered paraformaldehyde (4% *w*/*v*) for a minimum of 24 h at room temperature. Following fixation, samples underwent systematic dehydration using a graded ethanol series beginning with 75% ethanol for 4 h, followed by 85% ethanol for 2 h, 90% ethanol for 2 h, and 95% ethanol for 1 h. The dehydration process continued with absolute ethanol I for 30 min and absolute ethanol II for 30 min, concluding with alcohol-benzene clearing for 5–10 min. Paraffin infiltration was performed using three sequential wax baths at 65 °C, with each bath lasting 1 h. Tissues were embedded in paraffin blocks using automated embedding systems (Wuhan Junjie Electronics Co., Ltd., Wuhan, Hubei, China) and cooled on a −20 °C cryoplatform until solidification.

#### 2.3.3. Sectioning and Staining Procedures

Serial sections of 4 μm thickness were prepared using a rotary microtome (Leica RM2016) (Shanghai Leica Instruments Co., Ltd., Shanghai, China). Sections were floated on 40 °C water baths, mounted on positively charged glass slides, and dried at 60 °C until paraffin melted. Hematoxylin and eosin (H&E) staining was performed according to standard protocols, beginning with deparaffinization and rehydration. This process involved treatment with environmentally friendly dewaxing solution I for 20 min, followed by environmentally friendly dewaxing solution II for 20 min, absolute ethanol I for 5 min, absolute ethanol II for 5 min, 75% ethanol for 5 min, and a distilled water rinse.

The staining protocol commenced with HD H&E pre-treatment solution for 1 min, followed by hematoxylin staining for 3–5 min. Sections were then rinsed with tap water and differentiated, treated with bluing solution for color reversal, and washed with running water. The process continued with 95% ethanol treatment for 1 min and eosin staining for 15 s. Dehydration and mounting procedures involved sequential dehydration through graded alcohols and clearing agents, mounting with neutral balsam, and coverslip application.

### 2.4. Microscopic Analysis

Histological sections were examined using an upright optical microscope (Nikon Eclipse E100) (Nikon Corporation, Tokyo, Japan) equipped with a digital imaging system (Nikon DS-U3) (Nikon Corporation, Tokyo, Japan). Systematic evaluation was performed at multiple magnifications (40×, 100×, 400×) to assess epidermal architecture and stratification, dermal organization and cellular composition, presence or absence of cutaneous appendages, vascular and collagenous fiber distribution, and melanocyte distribution and density. Representative photomicrographs were captured at standardized exposure settings for comparative analysis between chestnut and normal skin tissues.

### 2.5. Statistical Analysis

Data analysis was performed using SPSS version 26.0 (IBM Corporation, Armonk, NY, USA). Descriptive statistics were calculated for all continuous variables, including means, standard deviations, and minimum and maximum values. Pearson correlation coefficients were computed to assess the relationships between chestnut dimensions and phenotypic traits (age, body weight, limb measurements, and vertebral counts).

Statistical significance was set at *p* < 0.05, with highly significant correlations defined as *p* < 0.01. Correlation matrices were visualized using heatmap representations generated with chiplot Online website (https://www.chiplot.online/ (accessed on 30 May 2025)). Data quality was verified through assessment of bilateral symmetry correlations and biological plausibility of observed relationships.

All statistical assumptions were tested prior to analysis, including normality assessment using Shapiro–Wilk tests and homogeneity of variance evaluation using Levene’s tests where appropriate.

## 3. Results

### 3.1. Morphological Characteristics of Chestnuts

The anatomical distribution of chestnuts (ergots) in donkeys demonstrates species-specific characteristics that distinguish them from their equine counterparts. In donkeys, chestnuts are exclusively located on the medial aspect of the proximal forelimbs, representing a significant anatomical divergence from horses, where these keratinous structures are present on all four limbs.

Comparative morphological analysis reveals that equine chestnuts exhibit considerable phenotypic variability in shape and demonstrate pronounced protrusion from the limb surface. Accurate dimensional quantification of these structures necessitates the implementation of specialized casting methodologies to obtain precise morphometric data [1].

**Figure 2:**
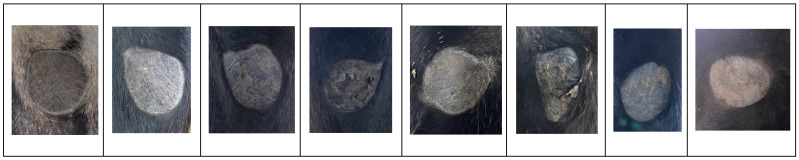

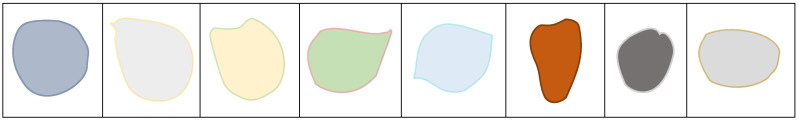
The different shape of chestnut. Most of them are regular ellipses, but there are still many irregular shapes that can serve as an identity mark for donkeys.

Morphological examination of chestnuts in Dezhou donkeys revealed distinct phenotypic characteristics. The majority of observed specimens presented regular geometric configurations, predominantly circular or elliptical in shape, with only a small proportion exhibiting irregular morphologies. These anatomical structures are characterized by the absence of hair follicles and display enhanced melanin pigmentation, resulting in visibly darker coloration compared to adjacent dermal tissues. This differential pigmentation pattern creates distinctive visual demarcation that facilitates clear identification and differentiation from surrounding anatomical structures (Figure 2).

The consistent morphological patterns observed in this study population suggest that chestnut characteristics may serve as reliable anatomical landmarks for species identification and potentially for individual identification within donkey populations.

### 3.2. Histological Characteristics of Chestnut and Adjacent Skin Tissues

#### 3.2.1. Epidermal Architecture

**Figure 3:**
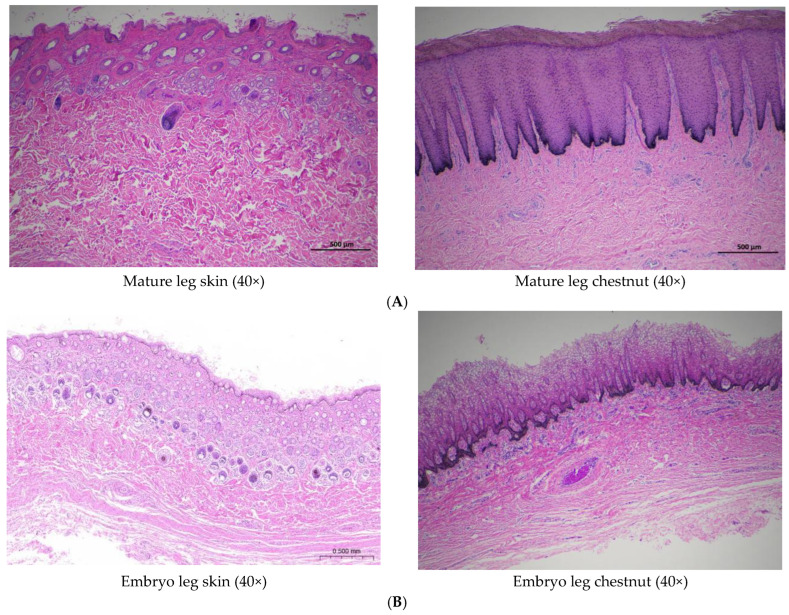

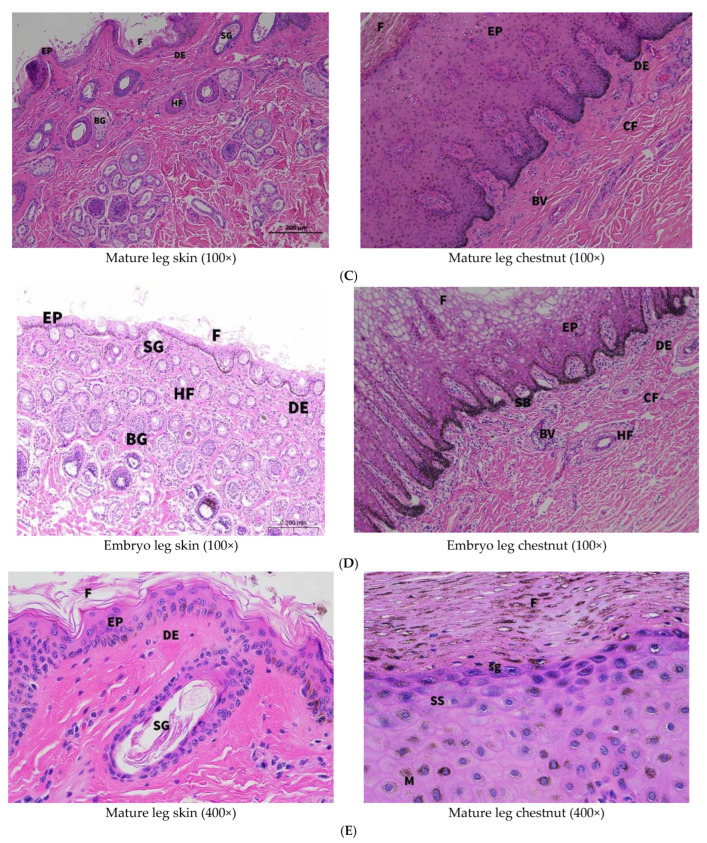

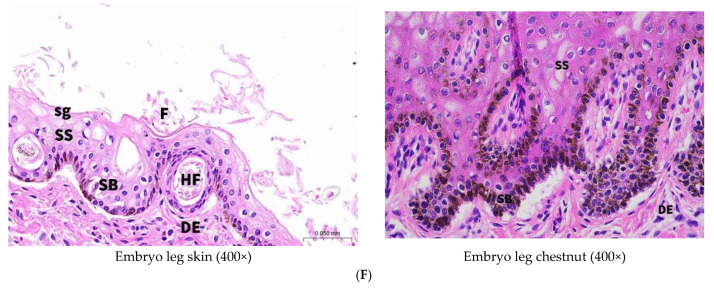
The skin tissue structure of Dezhou donkeys. (**A**) A skin tissue section 5 cm above the chestnut of the left front leg of an adult female donkey (40×). (**B**) A skin tissue section 5 cm above the chestnut of the left front leg of the embryonic male donkey (40×). (**C**) A skin tissue section 5 cm above the chestnut of the left front leg of an adult female donkey (100×). (**D**) A skin tissue section 5 cm above the chestnut of the left front leg of the embryonic male donkey (100×). (**E**) A skin tissue section 5 cm above the chestnut of the left front leg of an adult female donkey (400×). (**F**) A skin tissue section 5 cm above the chestnut of the left front leg of the embryonic male donkey (400×). Abbreviation: F: Stratum corneum; EP: Epidermis; DE: Dermis; BG: Sebaceous gland; SG: Sweat glands; CF: Collagen fiber; HF: Hair follicle; sg: Stratum granulosum; SS: Stratum spinosum; M: Melanocytes; BV: Blood vessels.

Comparative histological analysis revealed significant structural differences between the epidermis of normal leg skin and chestnut tissue in Dezhou donkeys (Figure 3). The epidermal thickness of normal leg skin ranged from 4 to 6 cellular layers, whereas chestnut epidermis demonstrated substantial hyperkeratosis with more than 30 cellular layers.

Both tissue types exhibited similar cellular composition, with keratinocytes representing the predominant cell population. These cells undergo continuous proliferation and differentiation, originating from the basal layer and migrating toward superficial layers through a well-characterized maturation process [10]. Microscopic examination revealed distinct morphological differentiation gradients in normal donkey skin, progressing from cuboidal or columnar basal keratinocytes through polygonal spinous layer cells and flattened granular layer cells, culminating in terminally differentiated, anucleated corneocytes in the stratum corneum. Concurrent nuclear and cytoplasmic modifications were observed throughout this differentiation process, with basal keratinocytes characterized by large, intensely basophilic nuclei, while stratum corneum cells demonstrated complete nuclear loss. Melanocytes containing melanin granules were sparsely distributed within the basal layer, consistent with photoprotective function [11].

In contrast, chestnut epidermis exhibited marked hyperkeratosis with a substantially thickened stratum corneum displaying advanced keratinization characteristics. The corneal layer demonstrated flattened cells with pyknotic or absent nuclei, eosinophilic cytoplasm, and significantly increased cellular stratification compared to normal skin. While normal skin exhibited a single granular layer of flattened, pre-keratinized cells, chestnut tissue contained 3–5 granular layers. Notably, melanocyte distribution in chestnut tissue extended throughout the entire epidermal thickness rather than being restricted to the basal layer. The pronounced accumulation of melanin granules within these melanocytes indicates enhanced pigmentation capacity, likely accounting for the characteristic dark coloration of chestnut tissue [12].

Morphologically, chestnut epidermis demonstrated more pronounced architectural definition compared to normal skin. The stratum corneum exhibited characteristic vacuolated morphology with absent nuclei and cytoplasm, while the granular layer contained polygonal cells that had not undergone complete flattening. The spinous layer displayed increased cellular density relative to both stratum corneum and stratum granulosum. Developmental analysis of 7-month-old fetal donkey skin revealed the presence of hair follicles and sebaceous glands within the epidermis, structures that were absent in adult specimens, while chestnut tissue consistently lacked these appendages throughout development.

#### 3.2.2. Dermal Architecture

The basement membrane zone, positioned at the dermal–epidermal junction, contains abundant extracellular matrix components that establish an adhesive microenvironment essential for maintaining structural integrity and stable interfacial adhesion between epidermal and dermal compartments [13]. The superficial dermis (papillary layer) connects to the epidermal basal layer via the basement membrane and contains densely arranged fine collagen fibers. The deeper reticular layer consists of thick, interwoven collagen fiber bundles and accommodates cutaneous appendages, including hair follicles, sweat glands, and sebaceous glands, in addition to vascular and neural structures [14].

Normal donkey skin exhibited single-unit hair follicular architecture with relatively high follicular density in the leg region. Sebaceous glands demonstrated well-developed morphology throughout the integument, though with reduced prominence in the leg region. Sweat glands, composed primarily of branched tubuloalveolar units, showed particular development in leg skin and were predominantly localized within the reticular dermis.

Conversely, histological examination of chestnut dermis revealed characteristic mesh-like interfascicular spaces within the dermal fiber network. Fibroblasts represented the predominant cellular component in these regions, responsible for synthesizing collagenous proteins, elastic proteins, extracellular matrix components, and various enzymatic factors [15]. While blood vessels and collagen fibers were clearly identifiable within the dermal layer, cutaneous appendages including hair follicles, sweat glands, and sebaceous glands were consistently absent.

Examination of fetal chestnut tissue demonstrated dense populations of collagenous and elastic fibers within the dermal layer, accompanied by occasional hair follicles and round or oval structures potentially representing developing sweat or sebaceous glands. These observations suggest that cutaneous appendages may undergo physiological regression during postnatal development, contributing to the specialized characteristics of mature chestnut tissue.

### 3.3. Age-Related Variations in Chestnut Morphometry

The study population encompassed animals ranging from 0.3 to 15 years of age, with a mean age of 5 years following exclusion of statistical outliers. The donkeys were grouped according to their adulthood. Pearson correlation analysis showed that the ages of the Dezhou donkey in Group A were positively correlated with the width of the chestnuts in the left and right forelimb (r = +0.527 *p* < 0.01; r = +0.500, *p* < 0.01). Conversely, no statistically significant correlation was observed between age and chestnut length parameters.

Notably, age demonstrated stronger positive correlations with hindlimb measurements compared to forelimb measurements, with the left hindlimb exhibiting the most pronounced relationship (r = +0.647, *p* < 0.01). In Group B, age had no significant correlation with all parameters. These findings suggest that chestnut width may serve as a reliable indicator of animal maturation, while length parameters remain relatively stable throughout the developmental period.

### 3.4. Correlations Between Chestnut Morphometry and Limb Dimensions

Morphometric analysis showed that in Group A, the average limb lengths of the forelimbs and hindlimbs were 73 cm and 84 cm, respectively. The development of the limbs was highly symmetrical. The correlation coefficients of the lengths of the left and right hindlimbs were 0.988, and that of the left and right forelimbs was 0.978. In Group B, the average limb lengths of the forelimbs and hindlimbs were 75 cm and 89 cm, respectively. The correlation coefficients of the lengths of the left and right hindlimbs were 0.963, and the correlation coefficients of the lengths of the left and right forelimbs were 0.915. These strong correlations indicate consistent bilateral development and the absence of significant limb asymmetry in the Dezhou donkey population, thereby validating the reliability of morphometric measurements.

The synchronous increase in forelimb and hindlimb dimensions conformed to established growth patterns, further supporting data integrity. Correlation analysis revealed significant positive associations between chestnut width and limb height measurements. In Group A, the strongest correlation was identified between left chestnut width and right hindlimb length (r = +0.634, *p* < 0.01). Additionally, a significant positive correlation was observed between age and limb length, particularly pronounced in the left hindlimb (r = +0.647, *p* < 0.01), indicating continued limb growth during the development process before the adult stage of animals ingroup. In Group B, chestnut width showed a significant positive correlation with length of the right forelimb. The strongest association was observed between the width of the left chestnut and the length of the right forelimb (r = +0.403, *p* < 0.01).

### 3.5. Body Weight Correlations with Chestnut Characteristics

Following statistical outlier exclusion, the mean body weight was determined to be 175.89 kg in group A, and that of group B was 295.05 kg. Correlation analysis revealed significant positive relationships between body weight and both chestnut length and width parameters. In Group A, body weight is significantly correlated with the wide parameter of chestnuts in left and right (r = +0.538, *p* < 0.01: r = +0.533, *p* < 0.01). In Group B, the body weight of donkeys was moderately significantly positively correlated with the wide parameter of chestnuts. Among them, the width of chestnuts on the right leg was most significantly correlated with body weight (r = +0.404, *p* < 0.01), indicating that the width expansion of chestnuts is more closely related to weight gain throughout the entire age range of donkeys.

These findings suggest that chestnut width measurements could serve as a practical morphometric indicator for animal selection in fattening programs. It is worth noting that in groups A and B, both body weight and limb height showed a significant positive correlation: in Group A, the strongest correlation with body weight was observed for right hindlimb length (r = +0.627, *p* < 0.01), whereas in Group B, body weight showed the highest correlation with left forelimb length (r = +0.499, *p* < 0.01). This relationship enhances the utility of limb measurement as a predictor of donkey population reconstitution.

### 3.6. Thoracolumbar Vertebral Configuration and Chestnut Morphometry

Vertebral enumeration revealed that Dezhou donkeys possess 17–19 thoracic vertebrae and 5–6 lumbar vertebrae. Among the five possible thoracolumbar combinations, the 18+5 configuration represented the predominant phenotype, comprising 64.29% of the study population [9].

In both Group A and Group B, the correlation analysis demonstrated no statistically significant associations between thoracolumbar vertebral number and either chestnut morphometric parameters or limb height measurements. Specifically, vertebral count showed no notable correlation with chestnut dimensions. In Group A, the number of thoracic vertebrae was weakly negatively correlated with the measured width of the right chestnut (r = −0.139, *p* > 0.05), though these relationships did not achieve statistical significance.

**Figure 4:**
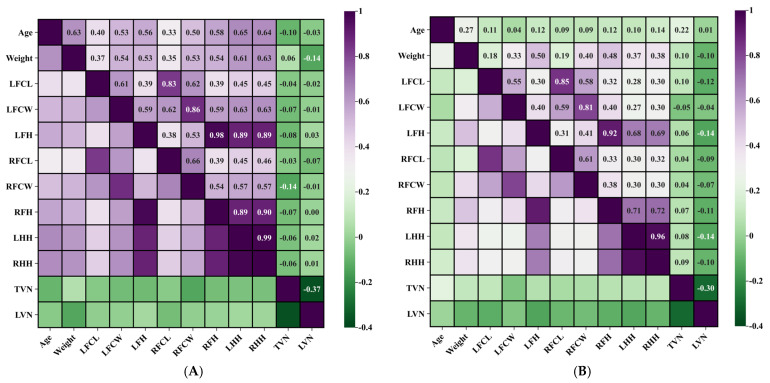
Chestnut correlation heatmap. (**A**) Heatmap of chestnut correlation analysis in Group A. (**B**) Heatmap of chestnut correlation analysis in Group B.

These findings indicate that skeletal axial variations in thoracolumbar configuration do not significantly influence appendicular morphometric characteristics, suggesting independent developmental regulation of these anatomical systems. Table 1 shows the minimum, maximum, and average values of all measurement indicators, while Table 2 and Table 3 shows the correlations among all the measurement indicators. Figure 4 indicates the heatmap visualization of the correlations among all measurement indicators

## 4. Discussion

The present study demonstrates that chestnuts in Dezhou donkeys and horses exhibit distinct morphological patterns characterized predominantly by regular geometric configurations. These findings support the potential utility of chestnuts as reliable biometric markers for individual identification, given their unique dimensional characteristics and consistent bilateral presentation. The morphological diversity observed across individuals, combined with the structural stability of these keratinized appendages, establishes a foundation for their application in equine identification systems.

Comparative histological analysis revealed fundamental structural differences between chestnut tissue and conventional donkey integument, providing crucial insights into the specialized nature of these structures. While normal donkey skin exhibited the expected tripartite organization with abundant cutaneous appendages and extensive vascular networks [16], mature chestnut tissue demonstrated highly specialized architecture. This specialization includes hyperkeratinized stratified squamous epithelium, widespread melanocyte distribution, and complete absence of typical skin appendages within the dermal compartment. The developmental examination of fetal specimens provides critical insights into chestnut ontogeny as the presence of vestigial cutaneous appendages in fetal tissues, followed by their regression during development, suggests that chestnut formation involves active involution of typical skin structures. This developmental pattern supports the hypothesis that chestnuts represent evolutionarily modified integumentary structures that have undergone specialized differentiation, potentially serving biomechanical or protective functions distinct from conventional skin.

The correlation analyses revealed intriguing species-specific patterns in chestnut growth dynamics. The positive correlation between chestnut dimensions and age in Dezhou donkeys aligns with previous observations in Arabian horses [1], suggesting conserved growth regulatory mechanisms across equine species. However, the contrasting patterns observed in Thoroughbred horses, particularly regarding hindlimb chestnuts, highlight species-specific developmental trajectories that warrant further investigation. The absence of hindlimb chestnuts in Dezhou donkeys compared to horses represents a significant anatomical difference that may reflect divergent evolutionary pressures or developmental constraints between these species. This variation underscores the importance of species-specific approaches when utilizing chestnuts for identification purposes.

Furthermore, the study reveals that chestnut development is integrated within broader somatic growth patterns, suggesting coordination through common regulatory mechanisms. The strong correlations observed between chestnut dimensions, body weight, and limb measurements indicate that these structures participate in overall musculoskeletal development rather than developing in isolation. The pronounced bilateral symmetry in limb measurements and synchronized appendicular development likely contribute to locomotor efficiency and biomechanical balance. This developmental coordination supports optimal gait mechanics and weight distribution, suggesting that even specialized structures like chestnuts may play roles in overall functional morphology.

Notably, the finding that thoracolumbar vertebral variation affects body morphology and tissue systems while remaining independent of chestnut dimensions provides important insights into developmental regulation. This independence reflects the distinct developmental origins and regulatory pathways governing dermal appendage formation versus axial skeletal morphogenesis, which operate through separate morphogenetic signaling cascades. This tissue-specific regulation suggests that chestnuts may be particularly useful as biometric markers precisely because their development is somewhat buffered from the general growth factors that affect overall body size and proportions, thereby maintaining their reliability as identification features across individuals with varying body conformations.

## 5. Conclusions

Altogether, our research successfully demonstrates that chestnuts in Dezhou donkeys are specialized integumentary structures with unique characteristics that distinguish them from normal skin and from horse chestnuts. Our study reveals that these structures exhibit hyperkeratosis, enhanced melanocyte distribution, and absence of cutaneous appendages, while showing strong positive correlations with age, body weight, and limb measurements. These findings support the potential utility of chestnuts as biometric markers for individual identification and indicate their integration within coordinated developmental programs.

Future research should expand sample diversity by including other donkey breeds to establish species-wide patterns and conducting longitudinal studies tracking individual animals over time to better understand growth dynamics. Practical applications could be developed through creating standardized protocols for chestnut-based identification systems, investigating the heritability of chestnut characteristics for breeding programs, and exploring machine learning approaches for automated chestnut pattern recognition. To deepen biological understanding, researchers should investigate the molecular mechanisms underlying chestnut development and melanocyte distribution, study the functional significance of chestnuts, and examine environmental factors that may influence chestnut morphology. Comparative studies across different equid species and investigations into evolutionary aspects of chestnut presence/absence patterns would provide valuable insights, while technical improvements, including non-invasive 3D imaging techniques, could enhance field applications.

Particularly important for advancing the field would be detailed molecular mechanism studies focusing on the differential gene expression patterns between chestnuts and normal skin tissue. Future research should investigate the role of key developmental pathways that regulate skin appendage formation and could explain the absence of hair follicles and glands in chestnuts. The enhanced melanocyte distribution observed in chestnut tissue warrants examination of melanogenesis-related molecular networks and their regulatory mechanisms. Additionally, studying the expression of keratinization-related molecular markers could elucidate the molecular basis of hyperkeratosis in chestnut tissue. Epigenetic mechanisms, particularly chromatin modifications and methylation patterns, should be analyzed to understand how the same genome produces such dramatically different skin phenotypes in adjacent tissue regions. Finally, investigating growth factor signaling networks could reveal how chestnut development becomes coordinated with overall somatic growth patterns, providing insights into the strong correlations observed between chestnut dimensions and body measurements.

## Figures and Tables

**Table 1:** Statistics of Dezhou donkey’s chestnut and body measurements.

	Age (year)	Weight (kg)	LFCL (cm)	LFCW (cm)	RFCL (cm)	RFCW (cm)	FLH (cm)	HLH (cm)
Minimum (group A)	0.3	79	2.0	2.2	2.4	2.4	57.5	64
Maximum (group A)	2	349	6.6	6.0	6.8	5.5	84	99
Mean (group A)	1.33	176.34	4.39	3.87	4.40	3.74	73.12	84.38
Minimum (group B)	2.5	212	3.1	3.2	3.2	2.9	66	78
Maximum (group B)	15	419	7.4	6.5	8.2	6.0	84	100
Mean (group B)	6.66	298.61	4.89	4.53	4.90	4.39	75.21	88.77

**Table 2:** Correlation Between Chestnut and Dezhou Donkey Phenotypes in Group A.

	Age	Weight	LFCL	LFCW	LFH	RFCL	RFCW	RFH	LHH	RHH	TVN	LVN
Age	1	0.632	0.397	0.527	0.563	0.333	0.5	0.576	0.647	0.643	−0.096	−0.03
Weight	0.632	1	0.374	0.538	0.53	0.353	0.533	0.543	0.614	0.627	0.057	−0.137
LFCL	0.397	0.374	1	0.612	0.387	0.835	0.622	0.389	0.45	0.451	−0.037	−0.017
LFCW	0.527	0.538	0.612	1	0.589	0.617	0.865	0.593	0.629	0.634	−0.074	−0.011
LFH	0.563	0.53	0.387	0.589	1	0.377	0.526	0.978	0.891	0.894	−0.08	0.026
RFCL	0.333	0.353	0.835	0.617	0.377	1	0.662	0.387	0.451	0.457	−0.031	−0.07
RFCW	0.5	0.533	0.622	0.865	0.526	0.662	1	0.539	0.568	0.572	−0.139	−0.014
RFH	0.576	0.543	0.389	0.593	0.978	0.387	0.539	1	0.892	0.895	−0.07	0.003
LHH	0.647	0.614	0.45	0.629	0.891	0.451	0.568	0.892	1	0.988	−0.057	0.02
RHH	0.643	0.627	0.451	0.634	0.894	0.457	0.572	0.895	0.988	1	−0.061	0.013
TVN	−0.096	0.057	−0.037	−0.074	−0.08	−0.031	−0.139	−0.07	−0.057	−0.061	1	−0.369
LVN	−0.03	−0.137	−0.017	−0.011	0.026	−0.07	−0.014	0.003	0.02	0.013	−0.369	1

**Table 3:** Correlation Between Chestnut and Dezhou Donkey Phenotypes in Group B.

	Age	Weight	LFCL	LFCW	LFH	RFCL	RFCW	RFH	LHH	RHH	TVN	LVN
Age	1	0.265	0.11	0.041	0.118	0.087	0.091	0.115	0.104	0.142	0.216	0.007
Weight	0.265	1	0.18	0.33	0.499	0.186	0.404	0.484	0.366	0.378	0.1	−0.099
LFCL	0.11	0.18	1	0.553	0.304	0.85	0.58	0.324	0.277	0.3	0.102	−0.12
LFCW	0.041	0.33	0.553	1	0.399	0.592	0.811	0.403	0.273	0.304	−0.051	−0.045
LFH	0.118	0.499	0.304	0.399	1	0.311	0.415	0.915	0.681	0.686	0.061	−0.144
RFCL	0.087	0.186	0.85	0.592	0.311	1	0.609	0.326	0.299	0.322	0.036	−0.091
RFCW	0.091	0.404	0.58	0.811	0.415	0.609	1	0.379	0.296	0.305	0.045	−0.074
RFH	0.115	0.484	0.324	0.403	0.915	0.326	0.379	1	0.709	0.722	0.074	−0.114
LHH	0.104	0.366	0.277	0.273	0.681	0.299	0.296	0.709	1	0.963	0.076	−0.137
RHH	0.142	0.378	0.3	0.304	0.686	0.322	0.305	0.722	0.963	1	0.09	−0.099
TVN	0.216	0.1	0.102	−0.051	0.061	0.036	0.045	0.074	0.076	0.09	1	−0.303
LVN	0.007	−0.099	−0.12	−0.045	−0.144	−0.091	−0.074	−0.114	−0.137	−0.099	−0.303	1

LFCL: left front limb chestnut length; LFCW: left front limb chestnut width; RFCL: right front limb chestnut length; RFCW: right front limb chestnut width; LFH: left front limb high; RFH: right front limb high; LHH: left hindlimb high; RHH: right hindlimb high; TVN: thoracic vertebrae number; LVN: lumbar vertebrae number. Correlations are not significant at the *p* > 0.05.

## Data Availability

All the data are available in the manuscript.
